# Exposures to Environmental Toxicants and Attention Deficit Hyperactivity Disorder in U.S. Children

**DOI:** 10.1289/ehp.9478

**Published:** 2006-09-19

**Authors:** Joe M. Braun, Robert S. Kahn, Tanya Froehlich, Peggy Auinger, Bruce P. Lanphear

**Affiliations:** 1 College of Nursing, University of Wisconsin-Milwaukee, Milwaukee, Wisconsin, USA; 2 Division of General and Community Pediatrics, Department of Pediatrics, Cincinnati Children’s Hospital Medical Center, Cincinnati, Ohio, USA; 3 Cincinnati Children’s Environmental Health Center, Department of Pediatrics, Cincinnati Children’s Hospital Medical Center, Cincinnati, Ohio, USA; 4 Division of Developmental Behavioral Pediatrics, Department of Pediatrics, Cincinnati Children’s Hospital Medical Center, Cincinnati, Ohio, USA; 5 Department of Pediatrics, University of Rochester School of Medicine, Rochester, New York, USA

**Keywords:** ADHD, attention deficit hyperactivity disorder, blood lead, children, environmental tobacco smoke, lead poisoning, NHANES, prenatal tobacco exposure, tobacco

## Abstract

**Objective:**

The purpose of this study was to examine the association of exposures to tobacco smoke and environmental lead with attention deficit hyperactivity disorder (ADHD).

**Methods:**

Data were obtained from the National Health and Nutrition Examination Survey 1999–2002. Prenatal and postnatal tobacco exposure was based on parent report; lead exposure was measured using blood lead concentration. ADHD was defined as having current stimulant medication use and parent report of ADHD diagnosed by a doctor or health professional.

**Results:**

Of 4,704 children 4–15 years of age, 4.2% were reported to have ADHD and stimulant medication use, equivalent to 1.8 million children in the United States. In multivariable analysis, prenatal tobacco exposure [odds ratio (OR) = 2.5; 95% confidence interval (CI), 1.2–5.2] and higher blood lead concentration (first vs. fifth quintile, OR = 4.1; 95% CI, 1.2–14.0) were significantly associated with ADHD. Postnatal tobacco smoke exposure was not associated with ADHD (OR = 0.6; 95% CI, 0.3–1.3; *p* = 0.22). If causally linked, these data suggest that prenatal tobacco exposure accounts for 270,000 excess cases of ADHD, and lead exposure accounts for 290,000 excess cases of ADHD in U.S. children.

**Conclusions:**

We conclude that exposure to prenatal tobacco and environmental lead are risk factors for ADHD in U.S. children.

Attention deficit hyperactivity disorder (ADHD) is one of the most common childhood disorders, with an estimated prevalence ranging from 3 to 8% [American Psychiatric Association (APA) 1994; Costello et al. 2003; Lesesne et al. 2003; Olfson et al. 2003; Pastor and Reuben 2002; Rowland et al. 2002; Wolraich et al. 1996]. Children who have ADHD are at increased risk for conduct disorder, antisocial behavior, and drug abuse later in life (Costello et al. 2003; Mannuzza 1993). Moreover, the costs associated with their medical care and education are substantial (Leibson et al. 2001). Although the mechanisms for the development of ADHD remain unclear, both genetic and environmental factors have been implicated (Ernst et al. 2001).

Numerous studies have found a significant association between prenatal environmental tobacco smoke (ETS) exposure and ADHD or ADHD-related behaviors, even after controlling for postnatal ETS exposures and familial psychopathology (Day et al. 2000; Fergusson et al 1993b; Kotimaa et al. 2003; Leech et al. 1999; Mick et al. 2002a; Milberger et al. 1996, 1998; Thapar et al. 2003; Wasserman et al. 2001; Williams et al. 1998). In case–control studies, investigators have found a 2- to 4-fold increased risk for ADHD associated with pre-natal ETS exposure (Mick et al. 2002a; Milberger et al. 1996, 1998). In contrast, the relationship between postnatal ETS exposure and children’s behavior problems has not been fully elucidated (Day et al. 2000; Fergusson et al 1993b; Weitzman et al. 1992; Williams et al. 1998). Moreover, the attributable fraction of childhood ADHD due to ETS exposure is unknown.

Environmental lead exposure, measured in blood or dentin, has been associated with higher rates of inattention and impulsivity (Bellinger et al. 1994; Fergusson et al. 1993a; Needleman et al. 1979; Wasserman et al. 1998, 2001). Although lead exposure is often cited as a risk factor for ADHD, existing studies examining the association of lead exposure with a diagnosis of ADHD, which were limited by small sample size, are inconclusive (David et al. 1972; Gittleman and Eskenazi 1983). Moreover, many of the studies examining the association of lead exposure with inattention or impulsivity involved children who had higher blood lead levels than the levels seen in contemporary children, and thus may not be directly relevant to children with lower levels of lead exposure (Bellinger et al. 1994; Needleman et al. 1979; Fergusson et al. 1993a; Wasserman et al. 1998, 2001). Although several recent studies have linked lead to IQ deficits at blood lead levels < 10 μg/dL (Bellinger and Needleman 2003; Canfield et al. 2003; Lanphear et al. 2005)—the current action level set by the Centers for Disease Control and Prevention (CDC)—it remains unclear whether blood lead levels < 10 μg/dL are also associated with behavioral problems in children (CDC 2005b).

Studies investigating the effect of environmental exposures on neurobehavioral outcomes have been complicated by evidence from both animal and human studies that toxicants may have differing effects in male and female subjects (Burns et al. 1999; Ernst 2001; McCartney 1999; O’Callaghan et al. 1992; Ribary and Lichtensteiger 1989; Ris et al. 2004; Weissman et al. 1999). The prevalence of ADHD is three times greater among males than among females (Costello et al. 2003; Lesesne et al. 2003; Olfson et al. 2003; Pastor and Reuben 2002). Some studies have documented varying behavioral effects of prenatal ETS exposure in males and females (Fergusson et al. 1998; Weissman et al. 1999), whereas other studies have not (Mick et al. 2002a; Milberger et al. 1996, 1998; Orlebeke et al. 1999). Studies of lead exposure’s effects have been similarly complicated: There is some evidence that males are at increased risk for externalizing behaviors (Burns et al. 1999) and attentional problems (Ris et al. 2004) from lead exposure, but the results are not entirely consistent.

The purpose of this study was to explore the relationship between exposure to ETS and environmental lead with ADHD using a large nationally representative sample of children. We also explored whether sex modifies the relationships between these neurotoxicants and ADHD. Finally we provide estimates of population attributable fraction of prenatal ETS exposure and lead exposure for ADHD in U.S. children.

## Methods

### Data source

The data for this analysis came from the National Health and Nutrition Examination Survey (NHANES), conducted from 1999 to 2002. The NHANES is a cross-sectional household survey of the noninstitutionalized civilian population. NHANES used a complex, multistage probability sampling design, with oversampling of children (< 5 years of age), Mexican Americans, and non-Hispanic blacks (CDC 2002a). Details regarding interviews, examination procedures, and sample collection have been described elsewhere (CDC 2002b).

### Assessment of ADHD and exposures to environmental toxicants

The primary dependent variables that we used to assess ADHD status were parent report of a diagnosis of ADHD and stimulant medication use. Parent-reported ADHD was based on the parent or guardian’s response to the question “Has a doctor or health professional ever told you that your child had attention deficit disorder?” To improve the specificity of the diagnosis of parent-reported ADHD, we used parent-reported ADHD and stimulant medication use as the main outcome. Stimulant medication use was based on National Drug Codes 03700, 17900, 39500, and 82000 (CDC 2005a). These codes correspond to amphetamine aspartate/amphetamine sulfate/dextroamphetamine aspartate/dextroamphetamine sulfate, dextroamphetamine sulfate, methylphenidate hydrochloride, and unspecified ADHD medication, respectively. Children who had only stimulant medication use or parent report of ADHD were excluded from the primary analysis. Secondary analyses investigated report of ADHD diagnosis and ADHD medication use as separate outcomes to confirm the validity of our primary analysis results.

We used parent report to measure children’s exposure to tobacco products. Measurement of prenatal ETS exposure consisted of the question “Did the child’s biological mother smoke at any time while she was pregnant with him/her?” No information on the quantity or brand of cigarettes smoked during pregnancy was collected. We assessed postnatal ETS exposure using parent-reported exposure to household ETS by asking “Does anyone who lives here smoke cigarettes, cigars, or pipes anywhere inside this home?” In a secondary analysis, we explored using the child’s serum cotinine concentration, a metabolite of nicotine, as a biomarker of ETS exposure (Bernert et al 2000). All children with cotinine values < 0.05 ng/mL were imputed from the left tail of the log-normal distribution using Cohen’s method (Cohen 1959). We focused primarily on reported presence of a smoker in the home as the independent variable because serum cotinine values were missing for 921 children.

We determined blood lead concentration by graphite furnace atomic absorption spectrophotometry (Miller et al. 1987; Parsons and Slavin 1993). The limit of detection was reported to be 0.3 μg/dL: 48 children had blood lead levels below this threshold. Nondetectable values were given values of 0.2 (0.3 divided by √2).

### Covariates

We examined multiple covariates and potential confounders for the association of prenatal ETS exposure and lead exposure with ADHD. Demographic variables included the child’s age, sex, race, and socioeconomic status [as measured by poverty-to-income ratio (PIR)]. PIR is the ratio of family income to the poverty threshold for the year of the interview. Children with PIR values < 1 are considered to be living below the poverty level. Health insurance coverage was also included as a covariate. In addition, a review of the literature suggested that preschool attendance, low birth weight, and ferritin levels (an indicator of iron status) should be considered potential confounders because of their prior documented associations with child behavioral problems and environmental toxicants [Knopik et al. 2005; Kordas et al. 2004; Mick 2002b; National Institute of Child Health and Human Development (NICHD) 2003; St. Sauver et al. 2004; Wasserman et al. 2001]. Child’s birth weight and admission to a neonatal intensive care unit (NICU) were included as markers of perinatal distress.

### Statistical analysis

We used logistic regression analysis with a binary outcome of ADHD to identify predictors of ADHD. Variables found to be associated with ADHD based on chi-square (*p* < 0.2) in bivariable analyses were included in the logistic regression analyses. Postnatal ETS exposure was forced into all multivariable models. Because NICU admission and birth weight may be acting as intervening variables on the pathway from prenatal ETS exposure to ADHD, we included these two variables in secondary analyses to examine whether their inclusion altered our findings (Kiely 1991). We also examined the relationship of lead exposure and ADHD at blood lead levels ≤ 5 μg/dL in secondary analyses.

Among children 4–15 years of age, 5,171 were available for analysis. We found that children who did not have routine access to health care were unlikely to be treated with stimulant medications; therefore, we excluded these children from the main analysis (*n* = 458). Regression diagnostics were conducted to identify influential observations and collinearity. Influential observations were excluded from analyses to examine whether their inclusion altered the results (*n* = 9). The exclusion of these outliers did not significantly influence the estimates of prenatal ETS exposure or environmental lead exposure. After excluding outliers and children without routine access to health care, 4,704 children were available for bivariate analyses; of those children, 3,879 had complete data available for multivariate analyses.

After developing a multivariable main effects model, we tested for an interaction between sex and prenatal ETS exposure, and between sex and blood lead concentration. For prenatal ETS exposure, we first analyzed the complete sample and modeled the potential interaction using a variable with four categories: unexposed females (reference category), exposed females, unexposed males, and exposed males (Rothman 2002). We also tested whether a formal sex-by-exposure interaction term was statistically significant for blood lead concentration and prenatal ETS exposure. We calculated population-attributable fraction (PAF) for risk factors independently associated with ADHD using Miettinen’s formula (Hanley 2001). Because these independent risk factors are not mutually exclusive of other risk factors, we also estimated the PAF of children having either environmental lead and prenatal ETS exposures.

Analyses were performed using the SUDAAN statistical package to account for the multistage, complex sampling design (Research Triangle Institute 2004). Sample weights were applied according to the National Center for Health Statistics guidelines (CDC 2004) to produce accurate national estimates, adjusting for the oversampling of minorities and young children.

This study was approved by the National Center for Health Statistics Institutional Review Board, Cincinnati Children’s Hospital Medical Center Institutional Review Board, and the University of Milwaukee College of Nursing Institutional Review Board. Consent was obtained from all participants.

## Results

Of the 4,704 eligible children 4–15 years of age, 344 (8.2%) had only parent-reported ADHD and 154 (4.3%) reported stimulant medication use, equivalent to 3.8 million and 2.0 million U.S. children and adolescents, respectively. Of the 4,704 children, 135 (4.2% weighted percent) had parent report of both ADHD and stimulant medication use, equivalent to 1.8 million children in the United States. (Table 1). In bivariate analyses, we found a significant association between parent-reported ADHD and stimulant medication use with prenatal ETS exposure (*p* = 0.023), preschool attendance (*p* = 0.003), male sex (*p* < 0.001), increasing age in years (*p* < 0.001), and health insurance coverage (*p* < 0.001) (Table 1). Non-Hispanic white children were more likely than other racial groups to report ADHD (*p* = 0.001).

**Figure 1:**
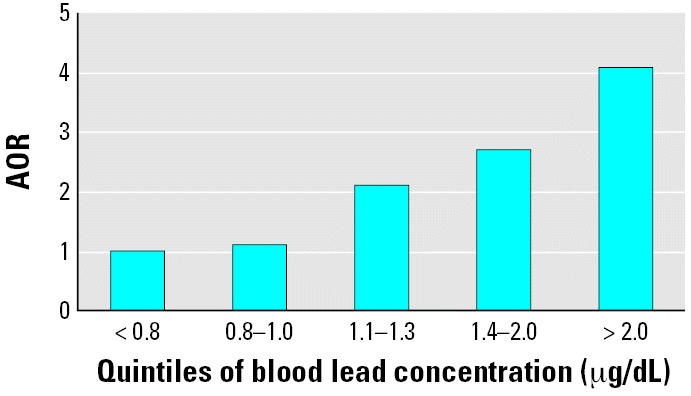
AOR for ADHD among U.S. children, NHANES 1999–2002, by blood lead concentration (μg/dL). The model was adjusted for child’s age, sex, race/ethnicity, preschool attendance, serum ferritin, prenatal ETS exposure, smoker in the household, and insurance status. *p*-value for trend = 0.012.

In multivariable analysis, prenatal ETS exposure and blood lead concentration were significant predictors of ADHD (Table 2). The adjusted odds ratio (AOR) for prenatal ETS exposure was 2.5 [95% confidence interval (CI), 1.2–5.2]. We also found a significant dose–response relationship between lead exposure and ADHD (Figure 1). Compared with children in the lowest quintile of blood lead concentration, children with blood lead levels in the fifth quintile (AOR = 4.1; 95% CI, 1.2–14.0) were at significantly higher risk for ADHD. The risk of ADHD was also significantly associated with male sex (AOR = 3.7; 95% CI, 2.1–6.6). Mexican-American and non-Hispanic black children had lower risks for reported ADHD diagnosis and stimulant medication use (AOR = 0.3; 95% CI, 0.1–0.7 and AOR = 0.5; 95% CI, 0.3–0.8, respectively) compared with non-Hispanic white children. Postnatal ETS exposure, as measured by the presence of a smoker in the home, was not a significant predictor of ADHD status in adjusted models (AOR = 0.6; 95% CI, 0.3–1.3; *p* = 0.224). The risk for ADHD was significantly associated with preschool attendance (AOR = 2.4; 95% CI, 1.1–5.1).

**Figure 2:**
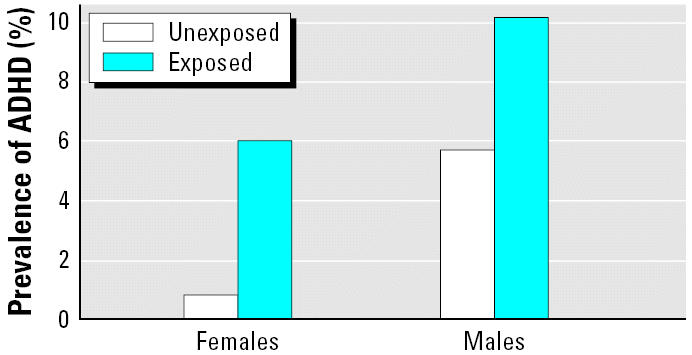
AOR for ADHD among U.S. children by prenatal ETS exposure and sex. The risk for ADHD among ETS-exposed children was greater in females; females who were prenatally exposed to tobacco were at 4.6-fold higher risk for ADHD compared with unexposed females (OR = 4.6; 95% CI, 1.7–12.4), whereas exposed males were at 2-fold higher risk for ADHD compared with unexposed males (OR = 2.1; 95% CI, 0.9–4.7) (*p* = 0.141 for sex by prenatal ETS exposure interaction). Model adjusted for race/ethnicity, sex, age, blood lead level, ferritin level, presence of a smoker in the home, preschool attendance, and insurance status.

Next, we tested for interactions between sex and prenatal exposure to ETS, sex and blood lead concentration, and prenatal exposure to ETS and blood lead concentration. We did not find a significant interaction between prenatal ETS exposure and sex using a formal interaction term (*p* = 0.141). Compared with unexposed females, females who were pre-natally exposed to ETS were at a 4.6-fold higher risk for ADHD compared with unexposed females (OR = 4.6; 95% CI, 1.7–12.4), whereas exposed males were at an almost significant 2-fold higher risk for ADHD than unexposed males (OR = 2.1; 95% CI, 0.9–4.7; *p* = 0.073) (Figure 2). There was not a significant interaction between blood lead levels by sex (*p* = 0.242) or blood lead levels by maternal smoking (*p* = 0.837).

We conducted secondary analyses to examine the effects of lead exposure at blood lead levels < 5 μg/dL and to test the stability of our results. When the sample was restricted to children with concurrent blood lead concentrations ≤ 5 μg/dL, there was still a significant association between higher blood lead levels and ADHD. Compared with children in the lowest quintile (nondetectable to 0.7 μg/dL), children with blood lead levels in the highest quintile (2.0–5 μg/dL) had a 4.5-fold (95% CI, 1.3–15.3) higher risk for ADHD.

When birth weight and NICU were added to the primary model, the adjusted OR for prenatal ETS exposure and the fifth quintile of blood lead levels did not change, remaining at 2.2 (95% CI, 1.0–5.1; *p* = 0.055) and 4.5 (95% CI, 1.3–15.6; *p* = 0.019), respectively. Consistent with postnatal ETS exposure measured by parent report, postnatal ETS exposure using serum cotinine was not associated with ADHD (AOR = 0.99; 95% CI, 0.97–1.00; *p* = 0.092). Using the same multivariable model, we found that when the ADHD outcome was defined simply by parent report or by stimulant medication use, rather than the combination of the two, the AOR of maternal smoking and blood lead level did not differ appreciably. Finally, there was no substantive change in the relationship of either prenatal ETS exposure or blood lead concentration when we included children without routine access to health care in the model.

The PAF for prenatal ETS exposure for both males and females was 18.4% (95% CI, 5.1–24.8%), corresponding to 270,000 cases of ADHD in children 4–15 years of age (Table 3). Although there was a significant dose–response relationship between lead exposure and ADHD, we estimated the population attributable fraction only for children who had blood lead levels in the fifth quintiles of blood lead concentration. Our estimates indicate that 21.1% (95% CI, 4.6–25.9%) of ADHD cases among children 4–15 years of age were attributable to having a blood lead > 2.0 μg/dL. This corresponds to 290,000 excess cases of ADHD among U.S. children 4–15 years of age (Table 3). Finally, we calculated the PAF for having either prenatal ETS exposure or blood lead concentration > 2.0 μg/dL to account for children who had both exposures. Our estimates indicate that 32.2% (95% CI, 4.2–41.3%) of ADHD cases among children 4–15 years of age were attributable to having either prenatal ETS exposure or blood lead > 2.0 μg/dL, which corresponds to 480,000 excess cases of ADHD among U.S. children 4–15 years of age.

## Discussion

Overall, 4.2% of children surveyed had parent-reported ADHD and were taking stimulant medication, equivalent to approximately 1.8 million U.S. children in the 4- to 15-year-old population. This rate is consistent with previous estimates of ADHD prevalence, which have ranged from 3 to 8% (APA 1994; Lesesne et al. 2003; Olfson 2003; Pastor and Reuben 2002). Our analysis confirms prior studies linking prenatal ETS exposure with ADHD and, for the first time, demonstrates a significant dose–response relationship between childhood lead exposure and ADHD. In contrast, we did not find a significant association between postnatal ETS exposure and ADHD.

In this sample, the overall adjusted risk for ADHD was 2.5-fold higher for children exposed prenatally to ETS, which is consistent with previous case–control studies (Mick et al. 2002a; Milberger et al. 1996, 1998). Previous studies using large cohorts have reported a 1.5-to 2.0-fold increase in risk for behavior problems among children whose mothers smoked during pregnancy, but they did not specifically examine ADHD status (Fergusson et al. 1993b; Weitzman et al. 1992; Williams et al. 1998). Using a large national sample, we confirmed that prenatal ETS exposure was a strong risk factor for ADHD, especially for females.

Although the difference in risks by sex was not statistically significant, these results suggest that females may be more susceptible to tobacco-associated ADHD. Females exposed prenatally to ETS had a 4.6-fold increased risk of ADHD compared with unexposed females, whereas males exposed prenatally to ETS were at a 2.1-fold increased risk for ADHD compared with unexposed males. When females are exposed prenatally to ETS, their risk of ADHD becomes equivalent to that of unexposed males.

Postnatal ETS exposure was not associated with ADHD in this analysis. We used parent report of household ETS and serum cotinine, but neither was associated with parent-reported ADHD. In previous research, investigators found a significant association of current tobacco consumption of the mother and externalizing behavior problems in children using continuous measures of behavior (Day et al. 2000; Weitzman et al. 1992; Williams et al 1998). It is possible that our results differ because we used a dichotomous outcome measure, thus limiting our sensitivity to detect an association between postnatal ETS exposure and ADHD-related behavior problems. Furthermore, the cross-sectional measures of reported postnatal ETS exposure and serum cotinine levels may have failed to detect postnatal ETS exposure at critical times in development that may be associated with ADHD or ADHD-related behaviors.

We found a significant dose–response relationship of higher blood lead levels and ADHD. Compared with the lowest quintile of blood lead levels, children with blood lead levels > 2.0 μg/dL were at a 4.1-fold increased risk of ADHD. When we limited the analysis to children with blood lead levels ≤ 5 μg/dL, the association between increased blood lead levels and ADHD remained. These results are consistent with previous reports that have found significant associations between blood or dentin lead levels and behavior problems (Bellinger et al. 1994; Fergusson et al. 1993a; Sood et al. 2001; Wasserman et al. 1998, 2001). Our results further indicate that blood lead levels below the CDC action level of 10 μg/dL are associated with an increased risk for ADHD in children (CDC 2005b). This result is consistent with previous studies that have found cognitive deficits in children with blood lead levels < 10 μg/dL (Canfield et al. 2003; Lanphear et al. 2000, 2005).

An interesting finding in this study was the increased likelihood of ADHD for children who attended preschool or child care. Presumably, children who attend preschool or child care are more likely to have problem behaviors noted by staff, thus increasing their chance for referral and diagnosis of ADHD by a health professional. Results from the NICHD child care study also suggest that children who are more aggressive and disobedient are more likely to be placed in child care at younger ages and for longer periods of time (NICHD 2003). Regardless of the reason for this finding, staff at these facilities may play an important role in identifying and referring children with behavior problems for early intervention.

This study has several limitations. First, the cross-sectional nature of our data makes it difficult to infer a causal relationship from our observed associations. Second, although some might argue that concurrent blood lead tests are not an adequate biomarker of a child’s lifetime exposure, recent studies indicate that concurrent blood lead level is a stronger predictor of lead-associated IQ decrements than blood lead measured during early childhood (Chen et al. 2005; Lanphear et al. 2005). Still, the stronger association between neurodevelopment and concurrent lead exposure has been observed only within cognitive domains and not behavioral domains. Third, although we were able to adjust for many important covariates and potential confounders, we were unable to adjust for others, such as maternal alcohol use during pregnancy and parental psychopathology.

An additional limitation of our cross-sectional data is susceptibility to recall bias. Mothers of children with behavior problems may be more likely to recall gestational intake of potentially harmful substances, such as tobacco, owing to a drive to identify a cause of their child’s disorder. On the other hand, mothers may fail to report tobacco use during pregnancy due to guilt or social stigma (i.e., social desirability bias). The reliance of our outcome variable on parent report is also a potential limitation. Nevertheless, by combining parent report of ADHD with stimulant medication use for our main outcome, we maximized the likelihood that all children counted as ADHD cases had been evaluated and diagnosed by a medical professional. On the other hand, these criteria required us to limit our analyses to children with routine access to health care services. Excluding children without routine access to health care enhances the specificity of the diagnosis of ADHD, but it also likely underestimates the total number of ADHD cases that are attributable to prenatal ETS exposure and environmental lead exposure.

Using a dichotomous measure of ADHD is less preferable than the administration of a DSM (*Diagnostic and Statistical Manual of Mental Disorders*)-based ADHD diagnostic instrument, which would offer increased specificity to detect behavior problems and specific ADHD subtypes, as well as the possibility of behavioral symptom counts. More continuous measures of ADHD symptoms or of specific underlying neurobehavioral domains may be the most powerful way to detect lead and tobacco effects, because ADHD may represent the tail of a continuum of behavioral effects produced by environmental chemical exposures and other etiologic factors (Mercugliano 1999). However, due to the considerable time and cost involved in the administration of DSM-based diagnostic instruments, previous national estimates of ADHD, such as the present investigation, have relied on medication use or parent report as measures of ADHD status (APA 1994; Lesesne et al. 2003; Olfson 2003; Pastor and Reuben 2002).

This study confirms the previously observed association of prenatal ETS exposure and ADHD. We also found a significant dose–response relationship between childhood lead exposure and ADHD. This analysis indicates that 270,000 ADHD cases in children 4–15 years of age are attributable to pre-natal ETS exposure, and 290,000 cases of ADHD among U.S. children 4–15 years of age are attributable to environmental lead exposure. The findings of this study underscore the profound behavioral health impact of these prevalent exposures, and highlight the need to strengthen public health efforts to reduce prenatal ETS exposure and childhood lead exposure.

## Figures and Tables

**Table 1:** Prevalence of stimulant medication use and parent-reported ADHD among children 4–15 years of age in NHANES 1999–2002 according to demographic and medical factors.

Variable	Sample size (*n*)	Weighted percent of parent-reported ADHD with prescription stimulant use (95% CI)	*p*-Value^a^
Total	4,704	4.2 (3.3–5.3)	
Age (years)			< 0.001
4–6	1,018	1.0 (0.4–2.4)	
7–9	1,003	4.8 (3.3–7.0)	
10–12	1,182	6.5 (4.5–9.2)	
13–15	1,501	4.5 (2.4–8.2)	
Sex			< 0.001
Male	2,264	6.6 (5.1–8.5)	
Female	2,440	1.8 (1.2–2.9)	
Race			0.001
Mexican American	1,519	1.0 (0.6–1.9)	
Other Hispanic	218	3.1 (1.4–6.8)	
Non-Hispanic white	1,293	5.5 (4.1–7.2)	
Non-Hispanic black	1,493	3.1 (2.2–4.4)	
Other, including multiracial	181	1.1 (0.3–4.1)	
PIR			0.639
1st quartile (0–1.04)	1,434	5.7 (3.6–8.9)	
2nd quartile (1.05–2.08)	1,121	3.6 (2.3–5.3)	
3rd quartile (2.09–3.73)	916	4.5 (2.6–7.7)	
4th quartile (3.74–5.0)	789	3.8 (2.7–5.5)	
Prenatal ETS exposure			0.023
No	4,014	3.4 (2.6–4.5)	
Yes	616	7.3 (4.8–11.2)	
Smoker in the home			0.210
No	3,669	3.9 (3.0–4.9)	
Yes	980	5.6 (3.5–9.1)	
Lead quintiles (μg/dL)			0.190
1st quintile (ND–0.7)	679	2.1 (0.9–4.7)	
2nd quintile (0.8–1.0)	795	3.4 (1.4–7.7)	
3rd quintile (1.1–1.3)	857	5.0 (3.6–6.9)	
4th quintile (1.4–2.0)	745	4.7 (3.1–6.9)	
5th quintile (≥ 2.0)	995	5.2 (2.9–8.9)	
NICU			0.504
No	4,129	4.0 (3.2–5.1)	
Yes	532	4.9 (2.9–8.2)	
Attended preschool			0.003
No	1,518	2.4 (1.3–4.5)	
Yes	3,178	4.8 (3.8–6.1)	
Covered by health insurance			< 0.001
No	630	0.2 (0.1–0.9)	
Yes	4,011	4.7 (3.7–6.0)	
Ferritin (ng/mL)			0.107
1st quartile (< 20)	965	2.7 (1.3–5.5)	
2nd quartile (20–29)	1,073	3.7 (2.5–5.5)	
3rd quartile (30–42)	955	3.8 (2.1–6.9)	
4th quartile (> 42)	1,017	7.7 (5.2–11.3)	
Birth weight			0.905
≥ 2,500 g	4,258	4.2 (3.3–5.4)	
< 2,500 g	393	4.0 (1.7–9.1)	
Cotinine tertiles (imputed) (ng/mL)			0.322
1st tertile (< 0.028)	1,288	3.4 (2.4–4.9)	
2nd tertile (0.028–0.259)	1,348	4.5 (2.7–7.6)	
3rd tertile (> 0.260)	1,183	5.8 (3.7–9.1)	

ND, Not detectable.

a Overall *p*-value for the variable.

**Table 2:** Logistic regression analysis for parent-reported attention deficit disorder among children 4–15 years of age, NHANES 1999–2002.^a^

Variable	AOR for parent-reported ADHD with prescription stimulant use (95% CI)	*p*-Value
Age (years)	1.1 (1.0–1.2)	0.016
Sex		
Female	Referent	
Male	3.7 (2.1–6.6)	< 0.001
Race		
Non-Hispanic white	Referent	
Other Hispanic	0.5 (0.1–2.1)	0.322
Mexican American	0.3 (0.1–0.7)	0.005
Non-Hispanic black	0.5 (0.3–0.8)	0.012
Other, including multiracial	0.2 (0.03–1.2)	0.072
Prenatal ETS exposure		
No	Referent	
Yes	2.5 (1.2–5.2)	0.020
Smoker in the home		
No	Referent	
Yes	0.6 (0.3–1.3)	0.224
Lead quintiles (μg/dL)		
1st quintile (ND–0.7)	Referent	
2nd quintile (0.8–1.0)	1.1 (0.4–3.4)	0.804
3rd quintile (1.1–1.3)	2.1 (0.7–6.8)	0.195
4th quintile (1.4–2.0)	2.7 (0.9–8.4)	0.086
5th quintile (> 2.0)	4.1 (1.2–14.0)	0.026
Preschool/child care attendance		
No	Referent	
Yes	2.4 (1.1–5.1)	0.022
Covered by health insurance		
No	Referent	
Yes	18.9 (3.7–97.4)	0.001
Ferritin (ng/mL)	1.006 (0.999–1.013)	0.089

ND, not detectable.

a Model adjusted for age, sex, race, prenatal ETS exposure, postnatal ETS exposure, blood lead levels, preschool or child care attendance, health insurance coverage, and ferritin levels.

**Table 3:** Population-attributable fraction of prenatal tobacco exposure and environmental lead exposure for parent-reported ADHD and stimulant medication use in children 4–15 years of age, NHANES 1999–2002.^a^

Characteristic	Exposed (%)	OR^b^	Attributable percent (95% CI)	Excess cases
Prenatal ETS exposure	30.7	2.5	18.4 (5.1–24.8)	270,000
Blood lead > 2.0 μg/dL	27.9	4.1	21.1 (4.7–25.9)	290,000
Prenatal ETS exposure or blood lead concentration > 2.0 μg/dL	46.2	3.3	32.2 (4.2–41.3)	480,000

a The risk factors are not mutually exclusive and the estimates of attributable risk are not additive. All ORs and attributable risks are adjusted for variables shown in Table 2.

b Model adjusted for age, sex, race, prenatal ETS exposure, postnatal ETS exposure, blood lead levels, preschool or child care attendance, health insurance coverage, and ferritin levels. Children without routine access to health care were excluded from the analysis.
